# YouTube Videos Related to the Fukushima Nuclear Disaster: Content Analysis

**DOI:** 10.2196/26481

**Published:** 2021-06-07

**Authors:** Limeng Cui, Lijuan Chu

**Affiliations:** 1 Department of Radiation Protection Beijing Center for Disease Prevention and Control Beijing Research Center for Preventive Medicine Beijing China; 2 Department of Drug Research Beijing SPXD-Pharm Research Corporation Limited Beijing China

**Keywords:** YouTube, Fukushima nuclear disaster, social media, risk communication, disaster, video platform, radiation, public safety, nuclear disaster

## Abstract

**Background:**

YouTube (Alphabet Incorporated) has become the most popular video-sharing platform in the world. The Fukushima Daiichi Nuclear Power Plant (FDNPP) disaster resulted in public anxiety toward nuclear power and radiation worldwide. YouTube is an important source of information about the FDNPP disaster for the world.

**Objective:**

This study's objectives were to examine the characteristics of YouTube videos related to the FDNPP disaster, analyze the content and comments of videos with a quantitative method, and determine which features contribute to making a video popular with audiences. This study is the first to examine FDNPP disaster–related videos on YouTube.

**Methods:**

We searched for the term “Fukushima nuclear disaster” on YouTube on November 2, 2019. The first 60 eligible videos in the relevance, upload date, view count, and rating categories were recorded. ﻿Videos that were irrelevant, were non-English, had inappropriate words, were machine synthesized, and were <3 minutes long were excluded. In total, 111 videos met the inclusion criteria. Parameters of the videos, including the number of subscribers, length, the number of days since the video was uploaded, region, video popularity (views, views/day, likes, likes/day, dislikes, dislikes/day, comments, comments/day), the tone of the videos, the top ten comments, affiliation, whether Japanese people participated in the video, whether the video recorder visited Fukushima, whether the video contained theoretical knowledge, and whether the video contained information about the recent situation in Fukushima, were recorded. By using criteria for content and ﻿technical design, two evaluators scored videos and grouped them into the useful (score: 11-14), slightly useful (score: 6-10), and useless (score: 0-5) video categories.

**Results:**

Of the 111 videos, 43 (38.7%) videos were useful, 43 (38.7%) were slightly useful, and 25 (22.5%) were useless. Useful videos had good visual and aural effects, provided vivid information on the Fukushima disaster, and had a mean score of 12 (SD 0.9). Useful videos had more views per day (*P*<.001), likes per day (*P*<.001), and comments per day (*P*=.02) than useless and slightly useful videos. The popularity of videos had a significant correlation with clear sounds (likes/day: *P*=.001; comments/day: *P*=.02), vivid information (likes/day: *P*<.001; comments/day: *P*=.007), understanding content (likes/day: *P*=.001; comments/day: *P*=.04). There was no significant difference in likes per day (*P*=.72) and comments per day (*P*=.11) between negative and neutral- and mixed-tone videos. Videos about the recent situation in Fukushima had more likes and comments per day. Video recorders who personally visited Fukushima Prefecture had more subscribers and received more views and likes.

**Conclusions:**

The possible features that made videos popular to the public included ﻿video quality, videos made in Fukushima, and information on the recent situation in Fukushima. During risk communication on new forms of media, health institutes should increase publicity and be more approachable to resonate with international audiences.

## Introduction

The Great East Japan Earthquake (magnitude 9.0) and subsequent tsunami on March 11, 2011, caused an accident at the Fukushima Daiichi Nuclear Power Plant (FDNPP). ﻿The FDNPP (location: 37° 25′ North, 141° 02' East) is located approximately 200 km northeast of Tokyo. This disaster released massive amounts of radioactive material into the environment [1] and resulted in public anxiety toward nuclear power and radiation worldwide [2,3]. Fukushima City will host 6 softball games and 1 baseball game for the Tokyo Olympic games [4], and food from Fukushima Prefecture will be served. Almost 9 years after the nuclear disaster, environmental and food safety issues are again drawing worldwide attention [5,6].

Social media can be defined as interactive communication media that have been fused into human lives worldwide [7,8]. YouTube (Alphabet Incorporated) has become the most popular video-sharing platform worldwide and is the second most visited website, with 2 billion monthly users [9,10]. Younger generations in particular are being raised in a time of social media and are learning to acquire information from these media. Previous studies have shown that YouTube can create a platform for and play a positive or negative role in risk communication [7].

Anxiety toward nuclear power is ﻿an important social issue. The public lacks knowledge about radiological issues and distrusts information provided by authorities [11]. They need multiple sources of information after the nuclear disaster. Therefore, cooperation among authorities, stakeholders, specialists, international organizations, traditional media, social media, and other networks is required for effective risk communication [12,13]. YouTube is thought to be an important source of information about the FDNPP disaster for the world. However, some studies in the medical and education fields have reported concerns about misleading information in YouTube videos [14-16]. Even information from commentators on large media platforms were ﻿full of hyperbole about the Fukushima nuclear disaster [17]. The massive amounts of information on social media that were posted after the disaster have also resulted in distrust toward authorities [18]. Sugimoto et al [19] found that rumors about disaster information from mass media were related to high fear of radiation.

This study aimed to examine the characteristics of YouTube videos related to the FDNPP disaster, analyze the content and comments of videos with a quantitative method, and determine which features contribute to making a video popular with audiences. 

## Methods

### Selection of Videos

**Figure 1:**
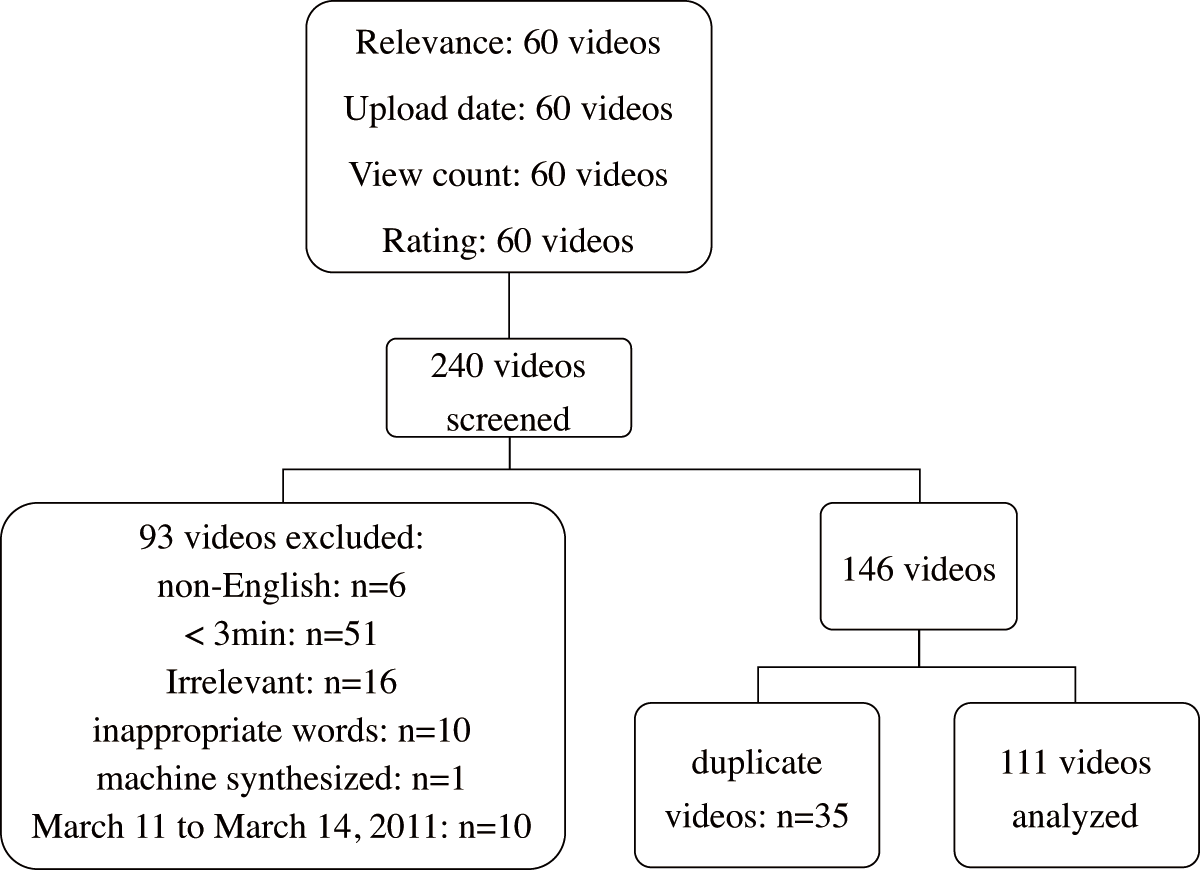
Flowchart showing the systematic video search and selection processes.

On November 2, 2019, videos were identified by using the search term “Fukushima nuclear disaster” on YouTube via the Google Chrome browser. YouTube sorts videos into the following four filter categories: relevance, upload date, view count, and rating. The first 60 eligible videos in each category were recorded (Figure 1). Videos that were irrelevant, were non-English, had inappropriate words, were machine synthesized, and were less than 3 minutes long were excluded. Videos that were less than 3 minutes long were found to be repeated summaries of news or reports of the nuclear accident. Therefore, these were excluded. Furthermore, videos released from March 11 to March 14, 2011, were excluded because they were mainly news that were reported during the emergency period of the disaster. In total, 146 videos met our inclusion criteria. After excluding 35 duplicate videos, 111 videos were analyzed.

### Collected Data

Characteristics of the videos, including the number of subscribers, length, the number of days since the video was uploaded, video popularity (views, views/day, likes, likes/day, dislikes, dislikes/day, comments, and comments/day), region, and affiliation (nonprofit organization or university, news source, for-profit company or organization, and private individual or layperson), were recorded (Table 1).

**Table 1:** Descriptive characteristics of videos.

Characteristics (N=111)	Value, mean (SD)	Value, median (range [minimum to maximum]; IQR)
Length (minutes)	23 (28)	12 (3-176; 31)
Number of days video was posted after the FDNPP^a^ disaster (March 14, 2011, to November 2, 2019)	1173 (1080)	898 (3-3154; 2047)
Number of authorship subscribers	1,522,000 (5,317,000)	15,000 (0-42,900,000; 851,000)
Total number of views	261,367 (619,315)	4826 (20-4,918,238; 241,671)
Number of views/day	632 (2388)	19 (0.04-16,34; 344)
Number of likes	2474 (5483)	53 (2-39,000; 2743)
Number of likes/day	27 (169)	0.4 (0-1625; 4)
Number of dislikes	298 (946)	298 (1-66,000; 173)
Number of dislikes/day	0.7 (2)	0.03 (0-15; 0.3)
Number of comments	533 (1042)	20 (0-6253; 766)
Number of comments/day	3 (15)	0.1 (0-135; 1)

^a^FDNPP: Fukushima Daiichi Nuclear Power Plant.

We divided videos into the following five types based on format parameters: news and interviews, formal presentations, informal presentations, educational videos, documentaries. The following parameters of videos were also recorded: whether Japanese people effectively participated in the video, whether the video recorder personally visited Fukushima, whether the video contained theoretical knowledge of radiation, and whether the video contained information about the recent situation in Fukushima (after January 2017).

The tone of the videos was coded as neutral and mixed or negative by two independent evaluators. No videos were coded as having a positive tone. Neutral and mixed videos were those that reported news or recorded the situation in Fukushima and interviews without personal comments and judgment. Negative videos presented conspiracy theories about Japan and described serious health risks to people in the world. The top 10 comments of each video were collected and divided into the following six categories by ﻿two independent evaluators: (1) conspiracy theories (eg, “Japan is not honest”); (2) serious health risks (eg, “Be careful with radioactivity. Especially avoid Fukushima foods”); (3) positive comments on the video (eg, “Great video. All the necessary details and easy to understand. I would like news reports to be like this, not the very superficial coverage that is usually available to the public”); (4) negative comments on the video (eg, “Is he going to actually talk about Fukushima or just himself?”); (5) negative comments on official agencies (eg, “The backup generators are built under the reactor. If they are outside it would have been avoided”); and (6) other (eg, “What a disaster”).

### Evaluation Tool

Based on previous studies on educational and medical videos [14,15,20-22], we modified our evaluation tool to identify useful, popular science videos about the Fukushima nuclear disaster. Useful, popular science videos provided scientifically correct and understandable knowledge, and its contents were acceptable to the public. The major criteria comprised the following: (1) content about radiation and the Fukushima disaster are scientifically correct; (2) the video is balanced and unbiased; (3) sounds are clear, and the background is free from noise; (4) images are clear; and (5) the video vividly captures the event and is engaging. The minor criteria comprised the following: (1) the video covers the topic identified; (2) the video is designed at the general audience level; (3) the creator or the organization providing the video is mentioned; and (4) information about the recent situation in Fukushima is provided.

Each item in the major criteria has two scores and in the minor criteria has one score [21]. Videos were categorized as useless (score: 0-5), slightly useful (score: 6-10), and useful (score: 11-14). Useful videos were well made and provided scientifically correct and unbiased information about the Fukushima disaster. ﻿Two independent evaluators who were knowledgeable about radiation protection and the Fukushima nuclear power plant accident scored each video. If the two evaluators' scores differed, reviewers discussed the videos in a meeting and reached an agreement.

### Statistical Analysis

Data were expressed as medians, minimums, and maximums. Normality was checked by using the Kolmogorov-Smirnov test. Since the variables were not normally distributed, nonparametric statistical tests were used. ﻿A Spearman correlation analysis was conducted to determine if video popularity correlated with the key parameters and scores. The Kruskal-Wallis test and Mann-Whitney U test were used to compare continuous variables. Statistical analysis was performed by using SPSS 21.0 (IBM Corporation). The significance level was set to *P*<.05.

## Results

### Characteristics of Videos

The descriptive information of 111 videos is summarized in Table 1. In total, 39 videos had a closed comment section or had no comments. Based on the posting dates, 11, 7, 13, 8, 3, 8, 12, 8, and 11 videos were uploaded on 2011, 2012, 2013, 2014, 2015, 2016, 2017, 2018, and 2019, respectively. The median duration of the videos was 12.1 minutes. Videos were posted from March 2011 to November 2019. The median number of views per day was 4826, and the number of views widely ranged from 20 to almost 5 million. Of the 111 videos examined, 109 (98.2%) had more likes than dislikes. The median number of comments per day was 0.12 (range 0-134.7).

The format of the videos, authorship type, and the nationality of video uploaders are shown in Multimedia Appendix 1. Of the 111 videos, 43 (38.7%), 41 (36.9%), 11 (9.9%), 9 (8.1%) and 6 (5.4%) videos were news and interviews, informal presentations, documentaries, formal presentations, and educational videos, respectively. No significant differences in video popularity (likes/day and comments/day) were found among the five video formats (likes/day: *P*=.17; comments/day: *P*=.38).

Private individuals and laypersons posted 40/111 (36%) of the videos, news sources posted 32/111 (29%) of the videos, nonprofit organizations or universities posted 19/111 (17%) of the videos, and for-profit companies or organizations posted 17/111 (15%) of the videos. News sources and for-profit companies or organizations had more subscribers than nonprofit organizations or universities and private individuals and laypersons. In addition to news sources, organizations and individuals uploaded different types of videos, including on-the-spot interviews, press conferences, news about the accident, wastewater treatment, explanations of the principles and processes of the nuclear accident, and personal opinions. However, only videos made by for-profit companies or organizations received more views per day than those made by nonprofit organizations or universities (*P*=.01) and laypersons (*P*=.03). Videos uploaded by private individuals and laypersons (*P*=.02), for-profit companies or organizations (*P*=.004), and news sources (*P*=.02) received more likes/day than nonprofit organizations or universities.

Of the 111 video uploaders, 45 (40.5%) uploaders were from the United States and only 14 (13%) uploaders were from Japan. Video posters from the United States received more subscribers, views per day, likes per day, and comments per day than those from Japan. Videos from other countries received more views per day (*P*=.01) and likes per day (*P*=.04) than those from Japan. In total, 60% (24/40), 21% (9/43), 18% (3/17), and 16% (3/19) of videos with a negative tone were uploaded by private individuals and laypersons, news sources, for-profit companies or organizations, and nonprofit organizations or universities, respectively.

### Useful Popular Science Videos About the Fukushima Nuclear Disaster

Of the 111 videos, 43 (38.7%) videos were useful, 43 (38.7%) were slightly useful, and 25 (22.5%) were useless (Table 2). The mean content score of the videos was 8.4 (SD 3.8). No significant difference was found between the content scores provided by the two evaluators (*P*=.99). Useful videos had good visual and aural effects, provided vivid information on the Fukushima disaster, and had a mean score of 12 (SD 0.9). ﻿The correlations between the total video scores and the number of subscribers, views per day, and likes per day were significant (*P*<.001). Useful videos had more views per day (*P*<.001), likes per day (*P*<.001), and comments per day (*P*=.02) than useless and slightly useful videos.

**Table 2:** Detailed characteristics of videos based on usefulness.

Characteristic	Useless (n=25), mean (SD; median; IQR)	Slightly useful (n=43), mean (SD; median; IQR)	Useful (n=43), mean (SD; median; IQR)	*P* value
Number of subscribers	1247 (6136; 1; 18)	921 (2412; 3; 50)	2282 (6759; 313; 1439)	<.001
Number of views/day	56 (161; 0.7; 47)	253 (599; 3; 298)	1345 (3700; 244; 603)	<.001
Number of likes/day	2 (5; 0.03; 1)	4 (8; 0.03; 4)	70 (282; 3; 6)	<.001
Number of dislikes/day	0.3 (0.6; 0.07; 0.5)	1 (2; 0.1; 1)	1.2 (3; 0.2; 0.6)	.37
Number of comments/day	0.5 (0.8; 0.1; 0.9)	1.4 (2.4; 0.4; 3)	8 (26; 0.6; 1)	.02

The popularity of videos had a significant correlation with clear sounds (likes/day: *P*=.001; comments/day: *P*=.02), vivid information (likes/day: *P*<.001; comments/day: *P*=.007), and understanding content (likes/day: *P*=.001; comments/day: *P*=.04).

### Key Parameters

**Figure 2:**
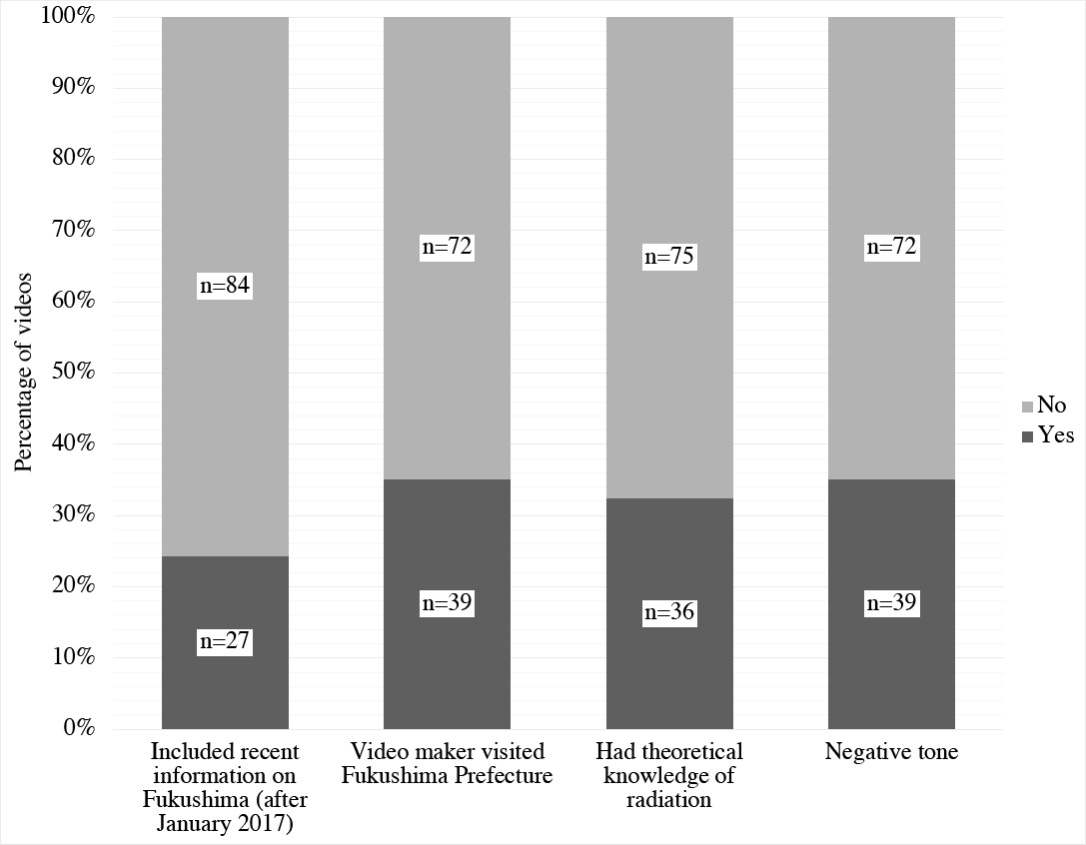
Distribution of the four key characteristics of videos.

We defined 4 key parameters after reviewing the videos (Figure 2). Videos about the recent situation in Fukushima had more likes per day (*P*=.01) and comments per day (*P*=.04). Video posters who made videos in Fukushima had more subscribers (*P*<.001), views per day (*P*=.003), and likes per day (*P*=.01). Videos with theoretical knowledge of radiation had more subscribers (*P*=.04) and dislikes per day (*P*=.02). There was no significant difference in likes per day (*P*=.72) and comments per day (*P*=.11) between negative and neutral- and mixed-tone videos.

### Comment Content Analysis

Of the 588 comments extracted from videos, 292 (49.7%) comments were negative and included conspiracy theories and criticisms of official agencies and the Japanese government. For example:

Their government said they would use rice from Fukushima for Olympic.

711 likes

Japan is lying to its people and neighbors. They lie that their radioactive food is safe.

659 likes

Positive comments on videos comprised 73 (12.4%) of comments. For example:

The older employees should be considered a hero. I believe nuclear plant workers who saved or slowed a devastating reaction should be internationally recognized as a hero.

5 likes

## Discussion

### Principal Findings

In this study, videos had a high number of views, likes, dislikes, and comments, indicating that viewers seek FDNPP-related information on YouTube. Private individuals and laypersons uploaded the greatest number of videos that provided information about personal experiences in Fukushima Prefecture, summaries of news, personal opinions, and their perspectives on the nuclear accident. Japanese health-related institutions’ videos comprised 12.6% (14/111) of FDNPP accident videos; this shows that these institutions did not pay sufficient attention to the YouTube platform in terms of the distribution of information overseas. It should be noted that no private individual and layperson uploaders were from Japan in this study. One of the reasons for this may be that only English videos were collected. Experts and the government should encourage people living in Fukushima Prefecture to engage with YouTube and show their real lives to the world.

Useful videos that had good quality and provided reliable radiation knowledge received more views, likes, and comments. The uploaders of these videos had more subscribers. The evaluation tool in this study was scientifically correct, balanced, and unbiased, but for the public, scientifically correct information and video tone were not statistically important elements of video popularity. This indicated the possibility of a large amount of misleading information being accessible to the public. A previous study also reported that governmental organizations' health information videos had limited impact [23]. The public has shown less interest in scientific educational videos and more in personal experiences [24]. The possible features that made videos popular to the public included the following: (1) the ﻿quality images and visuals, (2) sounds without background noises, (3) vivid and engaging content (4) understandable information, (5) videos made in Fukushima (personal experiences), (6) information on the recent situation in Fukushima, and (7) more subscribers.

In our sample of videos, educational and documentary videos that held a neutral position accounted for 15.3% (17/111) of the videos. Compared to the number of medical videos, there were fewer educational videos about nuclear accidents on YouTube [16]. One reason for this may be the cost and complexity of producing documentaries and educational videos. However, our analysis showed that various types of videos did not differ in popularity. Public health agencies may consider making simple videos, such as sightseeing and fieldwork recordings, to convey real-life situations in Fukushima Prefecture. The form of videos and the content they cover is not as important to the public as they are to experts. Instead of using complex scientific language, videos can use plain language to reach a wider audience in social media [18].

The most commonly watched videos were uploaded by for-profit companies or organizations, while the lowest number of likes per day was found on videos from nonprofit organizations or universities. The primary purpose of some videos was to attract viewers; thus, it is not surprising that the majority of video content was misinformation and conspiracy theories. Some studies have also reported on misleading health information on social media [23,25]. After the Fukushima nuclear accident, 80% of the total number of tweets on Twitter were created by 2% of influential accounts [26]. However, YouTube users can find varied videos when they conduct searches with keywords. Due to the difficulty of regulating YouTube videos and the platform's popularity, misleading videos may result in false impressions of the FDNPP disaster.

Our results show that half of the comments (292/588, 49.7%) were negative while the negative-tone videos comprised only 35.1% (39/111) of the total number of examined videos. For example, following a documentary uploaded by National Geographic, 1 comment said:

Fukushima was dangerous, is dangerous and will be dangerous.

We believe that videos that portrayed personal experiences elicited more engagement, as evidenced by the high number of views and likes and by the fact that videos describing the recent situation in Fukushima had more likes and comments.

### Limitations

This study has several limitations. First, only 1 search term—“Fukushima nuclear disaster”—was used, which might have resulted in videos on the study topic being missed. Second, we only analyzed videos in English and recorded publicly available metrics. In a follow-up study, it will be necessary to analyze Japanese videos of the Fukushima nuclear accident. Second, ours is a cross-sectional study that was conducted in November 2019; more videos have been made available since then. Last, although we used an evaluation tool, the subjectivity of judgment existed in this study. Conducting further research on content analysis of YouTube and improving the evaluation tools of popular science videos are necessary.

### Conclusions

This study is the first to examine the Fukushima nuclear disaster–related information specifically found on YouTube. ﻿A large amount of information on the Fukushima nuclear disaster is available on YouTube. In this cross-sectional study, 38.7% (43/111) of YouTube videos provided useful information about the Fukushima nuclear disaster. However, for the public, scientifically correct information and video tone were not statistically essential elements in video popularity. The possible features that made videos popular to the public included good ﻿technical design, understandable content, personal experiences, and publishers’ popularity. During risk communication on new forms of media, health institutes should increase publicity and try to be more approachable to resonate with international audiences.

## Appendix

Multimedia Appendix 1 Format of videos, authorship type and uploader nationality.

## Abbreviations

FDNPP: Fukushima Daiichi Nuclear Power Plant
