# Supplementary material for: YouTube Videos Related to the Fukushima Nuclear Disaster: Content Analysis
Source: JMIR Public Health Surveill. 2021 Jun 7;7(6):e26481. doi: 10.2196/26481 (PMC8218216; doi:10.2196/26481)
Supplement: Multimedia Appendix 1 [file publichealth_v7i6e26481_app1.docx]

**Multimedia Appendix 1**. Format of videos, authorship type and uploader nationality. ^a-b^

| **Format** | n (%) | Subscribers (k) | Range of dates posted | Views/day | Likes/day | Dislikes/day | Comments/day |
| --- | --- | --- | --- | --- | --- | --- | --- |
| News and interview | 43 (39) | 258 (0–12,500) | 1011 (4-3,145) | 156 (0.04-4,688) | 1 (0-48) | 0.2 (0.004-5) | 0.5 (0-14) |
| Formal presentation | 9 (8) | 15 (0-1,570) | 1277 (146-2,987) | 1 (0.3-847) | 0.005 (0.0008-5) | 0.02 (0.01-0.3) | 0.05 (0-1.5) |
| Informal presentation | 41 (37) | 1 (0-30,700) | 971 (3-3,154) | 3 (0.04-16,340) | 0.07 (0.003-1,625) | 0.2 (0.01-14) | 0.8 (0.002-135) |
| Educational | 6 (6) | 102 (3-42,900) | 2,181 (52-3,143) | 514 (1-9,874) | 1 (0.05-212) | 0.2 (0.008-5) | 0.5 (0.01-17) |
| Documentary | 11 (10) | 9 (0-130) | 1,000 (41-3097) | 19 (0.3-642) | 0.4 (0.001-5) | 0.1 (0.008-0.3) | 0.4 (0.02-2) |
| **Authorship** |  |  |  |  |  |  |  |
| Non-profit organization  or university | 19 (17) | 11 (0.07-1,570) | 1,650 (121-3,154) | 1 (0.08-940) | 0.003 (0.0008-5) | 0.08 (0.008-1) | 0.05 (0.0004-1) |
| News source | 32 (29) | 313 (0-7,730) | 771 (4-3,145) | 195 (0.06-882) | 1 (0.0008-34) | 0.2 (0.004-5) | 0.5 (0.005-10) |
| For-profit company or organization | 17 (15) | 103 (3-12,500) | 322 (52-2575) | 282 (0.3-9,874) | 3 (0.001-212) | 0.3 (0.01-5) | 1 (0.05-17) |
| Private individual/Layperson | 40 (36) | 2.3 (0-42,900) | 517 (3-3,152) | 3 (0.04-16,341) | 0.6 (0.0007-1,625) | 0.1 (0.01-14) | 0.2 (0.0003-135) |
| NA ^a^ | 4 (4) | 2 (0.3-8) | 2,604 (2,390-2,818) | 0.4 (0.1-0.7) | 0.001 (0.003-0.002) |  |  |
| **National** |  |  |  |  |  |  |  |
| Japan | 14 (13) | 3 (0.03-30,700) | 1,016 (3-3,154) | 0.5 (0.04-337) | 0.006 (0.0003-16) | 0.03 (0.01-0.2) | 0.005 (0.001-2) |
| US | 45 (40) | 82 (0-12,500) | 971 (15-3,152) | 113 (0.08-16,132) | 0.9 (0.0008-667) | 0.3 (0.004-14) | 0.9 (0.0003-59) |
| Others ^b^ | 35 (32) | 24 (0-5,410) | 564 (4-3,097) | 68 (0.2-16,341) | 2 (0.0007-1,625) | 0.2 (0.004-7) | 0.6 (0.001-135) |
| NA | 17 (15) | 0.7(0-42,900) | 777 (32-3,145) | 0.7 (0.2-619) | 0.07 (0.001-23) | 0.03 (0.01-1) | 0.4 (0.005-3) |

^a^ Values are median (minimum–maximum).

^b^ Australia, Belgium, Canada, Denmark, France, German, China, Italy, South Korea, Romania and United Kingdom; ^b^ No information.
